# 

**Published:** 1838-02-28

**Authors:** 


[SECOND EDITION.]

√

To the Editors of the MEDICAL EXAMINER.

GENTLEMEN—

The fifth number of your Journal contains the report of my lecture at the Philadelphia Hospital, delivered on the 3d of February, in which it became my serious duty to animadvert upon the danger and inutility of surgical operations in certain cases, as satisfactorily illustrated in a cruel experiment by my predecessor, in the attempted removal of a tumor, involving nearly the entire mouth and nares.

On the morning of the operation, Professor Gibson detailed to his class such arguments as he considered sufficient to authorize his surgical interference in so desperate a case. My arguments in opposition to the operation were not alluded to; he merely stated that his colleagues agreed with him as to the propriety of an operation, with the exception of Dr. Harlan. These arguments I took the liberty of laying before the students, at the subsequent lecture, the first of my course;—disclaiming all personality, I endeavoured to impress upon them the reasons for my unqualified disapprobation of such operations as the one in question. ⁜

"My learned and amiable friend," the Professor of Surgery, I am informed, has taken umbrage at the expression of an honest difference of opinion on this important point. He is indignant that any one out of the pale of the University, should be bold enough to doubt the infallibility of a professor before his pupils; it is too true that University opinions are already nearly a unit in the Medical Board of the Philadelphia Hospital, and the Professor is anxious to make them entirely so.

At the close of my lecture on Saturday last, Dr. Gibson made his appearance in the amphitheatre, *professedly* for the purpose of vindicating his conduct before the pupils; but his remarks, in reality, consisted in gross personalities and puerile witicisms towards myself, to the total exclusion of any argument tending to his vindication. I very much mistake the character of the majority of the Medical Class there convened, if they could find themselves in the least degree edified by the indelicate abuse in which he indulged on that occasion, so unworthy the dignity of a Professor of Surgery in the University of Pennsylvania.

The principal facts in my statements, and to the refutation of which his answer should have been confined, consisted mainly in this:—That the patient who had suffered so severely from the hammer, chisel, and red-hot pokers, had received no *permanent* benefit from these inflictions; and from the very nature of the tumor could not have been permanently benefited by any operation—that the patient, in fact, was as likely to die now from the tumor, as he was previously to the operation. This statement Dr. G. has not attempted to invalidate, notwithstanding his previous assertion, that the operation afforded the patient the only chance of life. Such being the nature of the observations of Dr. G., in his lecture of the 24th inst., I observed, with some regret and considerable mortification for the dignity of our profession, that you considered them worthy to be appended to the report of my lecture, in your last number, in which I never alluded disrespectfully to the operator, but confined my observations to the operation.

The nature of Dr. G.'s observations, destitute as they are of fact and argument, preclude the possibility of any notice on my part of a nature suited to the pages of your Journal, which ought always to be devoted to the dissemination of medical truths. Yet, were I disposed to indulge in invective, or to answer Dr. G. in his own strain—the theme is by no means a meagre one;—but I shall confine my remarks at present to a few hints, arising from his learned and witty discourse; which, if he be not incorrigible, may prove serviceable to him hereafter. I sincerely hope that the gentleman will not take offence at my candour—"for, knowing, as I well do, his extreme modesty, meekness, and humility, his tender sensibilities could not fail, I am sure, to be shocked by praise of this public description."

In the first place, Dr. G. entirely overlooked the fact, that his criticisms on my voice would apply, "*a fortiori*," to some of his colleagues, in whose success, as lecturers, his pecuniary interest is more involved. "Could it be otherwise," continues Dr. G., "that such a voice, loud and sonorous, peculiarly musical in its tone, clear and

⁜ the patient died 9th of march delightful in its cadénce, should fall upon your ear with all the harmony of sympathetic association." What possible bearing could such a personal allusion have upon the point at issue? We admit that Dr. G. possesses a very *loud* voice (to say nothing of its music)—but he is well aware that it is much easier to make a noise than to make sense in a lecture room. He should also know, that a simple truth, modestly told, is of far more importance than all the flowers of eloquence, or the pompous diction with which falsehood and error are occasionally clothed to make them palatable.

Dr. G. is peculiarly unfortunate, for one in his position, in his next attempt at a cut. "We feel peculiarly happy, gentlemen, in bearing testimony to the merits of our colleague, and have no doubt that the managers have retained his important services, in this great establishment, for the purpose of attracting to it full classes, such as I have the honour to be surrounded with at this moment."

It has been the subject of notorious remark, that the medical class attending these clinical lectures this season, does not amount to more than one half of the usual number, a falling off due to the insolent and ungentlemanly personality used by Dr. G., at the commencement of the Course, towards the students of the Jefferson Medical College, who almost to a man refused to honour him subsequently with their presence, preferring to take the Pennsylvania Hospital ticket—thus, by his ill-tempered and ill-timed personalities, defrauding the funds of the institution of at least one thousand dollars annually.

The learned Professor next takes much unnecessary pains to expose his own ignorance to his pupils, by pretending to despise those collateral sciences, the study of which has so much tended to elevate our profession. "Not only indeed is he (Dr. H.) distinguished in his particular vocation, but he has decided advantages over his less fortunate brethren, in being acknowledged, both in this country and in Europe, as profoundly acquainted with natural philosophy, natural history in all its branches," &c. Very different are the notions, on such matters, of the learned editors of the British and Foreign Medical Review, as is shown by the following extract from one of their recent numbers.

"The ungenerous notion formerly so successfully disseminated, that no medical man should attempt to be a man of science, exists no longer; *a medical man cannot preserve his intellectual rank in society without some scientific acquirement;* and subjects which were once confined almost to the closets of philosophers, are now discussed at dinner tables, declaimed upon in drawing-rooms, and settled at conversazione." The Professor would, consequently, find himself without intellectual rank in European society.

Professor G.'s own work on Surgery would have been more extensively useful, and a less fruitful source of error, had he paid some attention to the study of comparative physiology, comparative anatomy, and even zoology.

The success of the following attempted depreciation of my recent work, the "Medical and Physical Researches," is at least doubtful: "His large and splendid volume, too, on newts and frogs, toads and beetles, lizards and snakes, intermingled most naturally with aneurisms and tumors, fistulæ and strictures, &c., affords additional testimony of his industry and talents." Such unqualified praise, from such a pure source, might have been considered complimentary, had not numerous encomiastic criticisms of the work alluded to, both at home and abroad, rendered any observations of Dr. G. on the subject entirely supererogatory; and we further regret our inability to return the intended compliment, having searched in vain for any published opinion on his own work on Surgery beyond the Atlantic. But I am in possession of a written opinion, near at home, which I copied from the walls within the University, whilst his second volume was yet in press, the justness of which the modest Professor will not fail to acknowledge. It appears, that the medical students who subscribed to his work, were obliged to pay for both volumes on the delivery of the first; the second volume was delayed to an unreasonable period; the subscribers became restive, which produced re-iterated apologies from the Professor—under the impression, of course, that the acknowledged merits of number one, created the anxiety for the appearance of number two. The patience of the subscribers became, finally, exhausted. An Esculapian neophyte embodied the general sentiments of the class in the following couplet, written in mammoth letters on the wall near the entrance of the lecture room, with a piece of charcoal:—

"A student advises, whatever betides,
To leave the next volume alone—
The first is enough for our b———s,
The second, pray keep for your own!"

But for the terseness and truth of these lines, the fastidious reader might object to them on the score of delicacy; as it is, I think them worthy to descend to posterity, like the "fly in amber," embalmed by the surrounding medium; they are, besides, in strict unison with the Professor's familiar style with his pupils.

Dr. G. dwells on his great *surgical experience*, as in contrast with my own. As regards our public opportunities, we commenced our tour in the Philadelphia Hospital, the principal theatre of them, about the same period—say fifteen years ago. As respects the public hospitals of Europe, he visited them only as a student, to think with the brains of others: I enjoyed the advantage of examining those institutions with the freedom of a practical surgeon—receiving the honoured attentions of the veterans of science; and as far as private practice is concerned, since he came among us, the least successful practitioner, of equal age, would be loath to exchange with him.

Dr. G. endeavours to cast an imputation on me for visiting privately his patient, previously to the operation, when he knows it was my duty, as one of the surgeons in chief, to give my opinion on all cases involving capital operations; especially when requested so to do by the house-surgeon, by whom I was informed of the intention of Dr. G. to operate, in open violation of the articles governing such matters—and he was only enforced to consult his colleagues by an order from the managers.

This very important lecture on "*Tumor of the Antrum*," was further elucidated by Dr. G.'s informing the class of my "unrivalled *ornithological*" standing; and amused the class by dwelling on the fact, that the celebrated Audubon had named a new species of American bird, "FALCO HARLANI," or *Black Warrior!*—and concludes his transcendently witty and valuable *clinical* remarks, by a humble petition, that "our WARRIOR EAGLE, in all the nobleness of his nature, will 'still suffer little birds to sing.'"

The "little birds" are too well paid for singing, and too "snugly ensconced" behind the fortified walls of an invulnerable aristocracy, to be easily disturbed, even by a "war eagle's scream!" It is unreasonable, however, that they should desire to monopolize all the singing.

As the Professor appears interested in ornithology, I take the present occasion to communicate a fact in that science, from the knowledge of which he may draw consolation:—That classical ornithologist, Illiger, has classified certain birds of ignoble habits, under the denomination of CATHARTES, (including the turkey-buzzard;) these the "Warrior Eagle" holds in utter detestation, and never, *voluntarily*, associates with them—secure in their filthiness, they revel in their own excretions!

I might extend this letter to an indefinite length, were I disposed to recriminate. It would be easy to amplify upon the well known *candour* and *love of truth* for which the learned Professor is famous—the amiable, honourable, and magnanimous disposition he has ever displayed, in his intercourse with men, and which has secured to him the respect and esteem of so large a portion of his professional brethren without the sphere of the University of Pennsylvania, since he became a citizen of Philadelphia. The numerous foreign complimentary titles which have been conferred on him in acknowledgment of his industry, talents, and improvements in surgery!—the great bravery which he displayed, some years since, in boldly attacking a professor in a rival school, and his dexterity, wisdom, and discretion in obtaining a friend to fight his battle for him!—but, "*verbum sat*," and, for the present, I forbear.

This splendid specimen of the "*Clinical*" lectures, delivered in the Philadelphia Hospital, by the very popular Professor of Surgery in the University of Pennsylvania, taken in its literal sense, is no very great exaggeration of his every day style of discourse, both public and private; hence his obstreperous volubility on the present occasion.

Your much obliged, and very obedient, &c.,

R. HARLAN.

*Philadelphia, February* 28th, 1838.

*March* 5th, 1838.

"CATHARTES *Gibsoni*, is up again!"

Since the distribution of the first impressions of this letter, very numerous and urgent demands for copies, both in and out of the profession, render a second edition expedient.—I learn that the honourable Professor introduced the subject of this controversy, brought on by his own unprovoked and ungentlemanly attack, before his Class in the University of Pennsylvania, on Saturday last!—Surely it is a bird of ignoble habits that "befouls its own nest."

He commenced his lecture with a military flourish, and the ominous announcement that, the "Warrior Eagle is up again"—not doubting, it appears, of the success of his ineffectual attempt to *put me down.* The Professor, at least, is no judge in this matter. In reference to my letter, before his surgical class, the courageous Professor attempted no vindication of himself, nor any refutation of the grave imputations therein promulgated; but in lieu of which he regaled his hearers, with false misrepresentations and bilingsgate slang. His object is obviously to secure to himself an immunity by thus adopting a course of conduct which places himself beyond the pale of personal responsibility.

Abundant testimony exists to prove, that the assertions he made to his pupils, on the 3d inst., are utterly untrue, having no other foundation than in his own distempered imagination.

The imputations contained in my letter are not to be avoided by such contemptible subterfuges—these will adhere like "the shirt of Nessus," to this modern Hercules—this tunic can be exchanged only for the shroud.

